# Implementation of the milestones communication approach for patients with limited prognosis: evaluation of intervention fidelity

**DOI:** 10.1186/s12904-020-0527-1

**Published:** 2020-02-18

**Authors:** Jasmin Bossert, Michel Wensing, Michael Thomas, Matthias Villalobos, Corinna Jung, Anja Siegle, Laura Hagelskamp, Nicole Deis, Jana Jünger, Katja Krug

**Affiliations:** 1 Department of General Practice and Health Service Research, University Hospital Heidelberg, Im Neuenheimer Feld 130.3, 69120 Heidelberg, Germany; 2 Department of Thoracic Oncology, Thoraxklinik Heidelberg, Heidelberg University Hospital, Röntgenstraße 1, D-69126 Heidelberg, Germany; 3 Institute of Medical and Pharmaceutical Proficiency Assessment, Malakoff Passage, Rheinstraße 4, D-55116 Mainz, Germany

**Keywords:** Communication, Lung cancer, Advances care planning, Prognostic awareness, Patient preferences

## Abstract

**Background:**

Despite improvements in diagnostics and therapy, the majority of lung tumours are diagnosed at advanced stage IV with a poor prognosis. Due to the nature of an incurable disease, patients need to engage in shared decision making on advance care planning. To implement this in clinical practice, effective communication between patients, caregivers and healthcare professionals is essential. The Heidelberg Milestones Communication Approach (MCA) is delivered by a specifically trained interprofessional tandem and consists of four milestone conversations (MCs) at pivotal times in the disease trajectory. MC 1 (Diagnosis): i.e. prognosis; MC 2 (Stable disease): i.e. prognostic awareness; MC 3 (Progression): i.e. reassessment; MC 4 (Best supportive care): i.e. end of treatment. In between MCs, follow-up calls are carried out to sustain communication. This study aimed to assess to what extent the MCA was implemented as planned and consolidated in specialized oncology practice.

**Methods:**

A prospective observational process evaluation study was conducted, which focused on the implementation fidelity of the MCA. All MCs during two assessment periods were included. We analysed all written records of the conversations, which are part of the routine documentation during MCs and follow-up calls. Adherence to key aspects of the manual was documented on structured checklists at the beginning of the implementation of the MCA and after 6 months. The analysis was descriptive. Differences between the two assessment periods are analysed with chi-square tests.

**Results:**

A total of 133 MCs and 54 follow-up-calls (t1) and of 172 MCs and 92 follow-up calls (t2) were analysed. MC 2 were the most frequently completed conversations (*n* = 51 and *n* = 47). Advance care planning was discussed in 26 and 13% of MC 2 in the respective assessment periods; in 31 and 47% of MC 2, prognostic awareness was recorded. The most frequently documented topic in the follow-up calls was the physical condition in patients (82 and 83%).

**Conclusion:**

The implementation of a trajectory-specific communication concept was largely successful. Additional studies are needed to understand how fidelity could be further improved.

**Trial registration:**

DRKS00013469 / Date of registration: 22.12.2017.

## Background

Lung cancer remains the most prevalent cause of cancer-related deaths worldwide [1, 2]. In Germany, the overall age-adjusted 5-year relative survival is 16.9%, with considerable differences between men (15.5%) and women (20.3%) [3]. Cancer stage at the time of diagnosis is the most important predictor for treatment options, response to treatment and survival. Only 14% of patients are diagnosed in stage I, since lung cancer in early stages is often asymptomatic [4]. The majority of lung tumours is diagnosed at the advanced stage IV (men: 72.4%, women: 69.1%) with a poor prognosis compared to other cancer diseases [3], which is often associated with high psychological, physical and social stress for patients and family caregivers [5]. Due to the poor prognosis, patients need to be aware of the limited life span to effectively engage in end-of-life decision making on advance care planning (ACP) under consideration of patient preferences [6].

To meet the challenge of coping with the complex needs of patients [7], an effective communication from the time of diagnosis between all involved persons (patients, family caregivers and healthcare providers) is essential [8]. The opportunity to communicate about the situation positively affects patients’ quality of life [9]. Furthermore, communication is key to gathering important information, assisting patients in decision making and integrating palliative care early in the course of disease [10].

In spite of knowing about the positive effects of communication, patients, caregivers and healthcare providers often perceive communication skills of healthcare staff to be suboptimal and fragmented. Therefore, training of communication skills is recommended [7, 11] as well as a team approach to communication with patients, involving physicians and nurses. However, the actual application of communication skills in a team approach in routine practice remains difficult. It is therefore important to measure and improve its fidelity. The fidelity of any intervention is defined as the degree to which it is implemented as it was intended [12]. It reflects the adherence to content, frequency, duration and coverage of the planned approach [13]. Specific factors which are associated with intervention fidelity can be identified, such as aspects of environmental context, resources or the professional role understanding of healthcare professionals involved [14]. Insight into intervention fidelity and associated factors allows to tailor implementation strategies to the identified performance gaps and underlying causes [15].

For patients newly diagnosed with lung cancer stage IV, a structured longitudinal communication concept was developed and implemented to proactively address their complex care needs: The Heidelberg Milestones Communication Approach (MCA) [7]. MCA aims at providing coherent care that integrates palliative care early and across the disease trajectory [7]. The concept includes four types of so-called milestone conversations (MCs) which involve patients, caregivers and an interprofessional tandem of physician and nurse navigator. MCs are scheduled at specific pivotal times with a thematic focus in the disease trajectory: Disclosure of diagnosis and prognosis (MC 1), Treatment with response or stability of disease (MC 2), Disease progression with reassessment of options and change or stop of disease-modifying interventions (MC 3), and Transition to best supportive care and end-of-life consensus (MC 4). In follow-up sessions about a week after each MC, the nurse calls patients to sustain communication [7]. Content and implementation of the MCA are described in detail in the manual (available (in German) upon request from the authors). The implementation of a new communication approach in routine care faces the challenges of behavioral changes in health professionals, adaptation of procedures and processes both within an organization and outside/between organizations [16]. For implementing the MCA, behavioral changes in health professionals are addressed in a compulsory training on interprofessional communication tailored to the specific conditions of the health care team in training. The training takes place once a month over a period of four months and includes an introduction of the concept and an on-the-job video-feedback. A shared routine documentation shall improve communication within the team [7]. Furthermore, necessary organizational changes (e.g. time slots and rooms) are addressed in the institution to facilitate team-based communication with patients.

Studies have shown that newly introduced concepts are adapted to routine care after implementation, thereby (at least partly) not adhering to the concept aim. Aspects perceived as helpful in routine care are consolidated, others abandoned. An assessment of key aspects after a consolidation period may elucidate approaches for further improvement of a concept [17].

The aim of this study is to assess the fidelity of the MCA approach after these implementation strategies, a process evaluation was conducted. The process evaluation focuses on contents (documented topics) in the MCs according to the manual both at the beginning of the implementation and after a consolidation period. Furthermore, we documented the frequency of topics addressed in the conversations.

## Methods

The process evaluation was a prospective observational study with two assessment periods. Content analysis of patient files was used to evaluate implementation fidelity of the communication approach. All written records which are part of the routine documentation during MCs and follow-up calls were used for qualitative and quantitative analysis. Adherence to the manual was evaluated during the training period (t1: January to May 2018) and after six months (t2: September and October 2018). The first phase included five months, since there are fewer MCs conducted in the beginning that need to be integrated into clinical routine; the second phase included two months, when after implementation and routine use more MCs could be expected. Analysis focused on prognostic awareness, ACP and patient preferences.

### Setting

The study took place in 2017 and 2018 on the chemotherapy outpatient department at the Thoracic Clinic, University Hospital Heidelberg, Germany. This hospital is a comprehensive cancer center and focuses on thoracic diseases, including lung cancer.

#### Data collection

All routine records collected by nurses in the observed periods were included in the data analysis. The nurses as part of the interprofessional tandem are responsible for the documentation of the routine records during MCs and follow-up calls. Five nurse navigators for oncology and palliative care and five physicians have completed the compulsory tailored training for interprofessional communication based on the MCA manual between December 2017 and March 2018. The training included the topics: communication theories, breaking bad news, physician-patient communication, interprofessional communication, prognostic awareness, hope, SPIKES [18], NURSE [19] and shared decision making. The theoretical input was trained with simulated patients and as training on the job (observation and video feed-back of conversations with real patients). Additionally, the nurse navigators had a seven-day theoretical and practical briefing in October 2017, in which relevant aspects for nursing within the MCA were discussed. After the training, three nurse navigators conducted and documented the MCs together with physicians and the follow-up calls.

### Routine MC and follow-up call records

Routine records are used for MCs and follow-up calls. They are partly standardized thus allowing the nurse navigators to document individually in free text. The standardised parts include information on date and duration of MC or follow-up call. The routine record for MCs also includes information about participants (physicians, nurse navigators, and family caregivers), number of MC (1–4 or other conversations) and current disease status (stable, unstable, debasing, and dying). The records for follow-up calls additionally include information on the use of the Integrated Palliative Care Outcome Scale (IPOS) [20]. All aspects addressed by patients, physicians and nurses were documented in free text in the routine records. For analysis, contents of routine records were summarized on checklists for MCs (Additional file 1) and follow-up calls (Additional file 2). Checklists were qualitatively developed based on the aspects addressed in the manual of the MCA (Table 1).

**Table 1:** Specific topics of the MCA manual

MCs	Period in disease trajectory	Specific topics of the manual
I	Diagnosis	• Warn patient that there is bad news (SPIKES) • Open and honest statement of diagnosis and prognosis • Discuss further procedure (diagnostic, therapy) • Show limitations of therapy options
II	Stable disease	• Summarize last conversation • Clarify how patients think about the disease (Prognostic awareness) • Use stable disease to talk about advance care planning regarding end-of-life care. • Define precautionary authorized representative • Advance care planning can also be discussed later (depends of patient’s psychological condition)
III	Tumour progression	• Communicate tumour progression • Check patient preferences • Define new therapy goals regarding quality of life • Bring in palliative care offers (IPOS) • Question Prompt List
IV	Best supportive care	• Communicate the end of tumour centred treatment options • Transition to best supportive care • Include always patient preferences
General topics	In every conversation	• Organisation • Physical condition • Psychological condition • Therapy • Complementary medicine • Patient preferences

### Checklist for MCs

Data for analysis were extracted from the records using self-developed checklists. An aspect addressed in the free text part of the MC records was documented on the checklist as one of six general or of three MC-specific topics (Additional file 1).

“General topics of the MCA” include six aspects that are not linked to a particular MC, but are mentioned as either generally important (therapy, patient preferences, physical condition, psychological condition) or are not specifically described in the manual of the MCA (organisation, complementary medicine) (Additional file 1), but emerged from the MC records at the beginning of MCA implementation as frequently discussed. Additional file 3 (supplementary material) includes a detailed description of aspects and examples.

The MC-specific topics classify the process within the trajectory of a metastatic disease with a limited prognosis (Additional file 1). MC 1 focuses on patients’ understanding of the disease, thus, diagnosis, further diagnostics procedures and prognosis should be addressed. At MC 2, in the stable situation of the disease, prognostic awareness and ACP should be addressed to prepare for the case of progression. For MC 3, tumour progression and the facilitation of prognostic awareness are paramount. MC 4 focuses on the transition to best supportive care as all possible disease-modifying interventions are exhausted including patients preferences [11]. Additional file 3 (supplementary material) includes a detailed description of aspects and examples.

### Checklist for follow-up calls

For the checklist of follow-up calls no specific aspects are given in the manual, but the use of the Integrated Palliative Care Outcome Scale (IPOS) is recommended, which measures palliative care needs for patients and their families [20]. The checklist for follow-up calls initially consisted of the following standardised information: use of the Integrated Palliative Outcome Scale (IPOS), duration of the follow-up call, participating person, and date. The checklist was further developed based on frequently addressed aspects in the follow-up calls documented in the records at the beginning of MCA implementation. These aspects could be subsumed under six topics (Additional file 2) “Physical condition” and “Psychological condition” (both based on the IPOS), “Medical care”, “Nursing care”, “Outpatient providers”, and “Wishes and hopes” (emerged from records). Additional file 4 (supplementary material) includes a detailed description of aspects and examples.

#### Data analysis

All routine MC and follow-up call records were anonymized prior to analysis. Content of records was analysed quantitatively: For each record, incidences of aspects on the respective checklist were entered into a data matrix and further analysed using IBM SPSS version 24. Aspects were summarized to respective topics. A topic was considered as “addressed” if at least one aspect of the topic was documented. Absolute and relative frequencies of topics are reported for both assessment periods. For consolidation, frequencies of the main topics ACP, prognostic awareness and patient preferences in the two assessment periods were compared using Chi square tests.

## Results

### MCs

In the first assessment period (t1), 133 MCs were carried out: 27 MC 1 for disclosure of diagnosis and prognosis, 51 MCs 2 for the treatment with response or stability of disease, 30 MC 3 discussing disease progression with reassessment of options and change or stop of disease-modifying interventions, two MC 4 for the transition to best supportive care and end-of-life consensus, 14 other conversations without a special thematic focus and 9 conversations without a documentation of the MC (Table 2). The mean average duration of the MC was 30 min (SD = 10.7; range: 20–60 min). Each nurse navigator documented an approximately equal share of the routine records.

**Table 2:** Thematic focuses of ME – period comparison

	t1 (*n* = 133)	t2 (*n* = 172)	***p***-value/ direction change (increase/decrease)
**Milestone conversation (MC 1):**	**N (%)** **27 (100)**	**N (%)** **18 (100)**	
**Diagnosis**	6 (21)	9 (50)	.04 ↑
**Further diagnostics**	12 (43)	8 (44)	.92
**Prognosis**	5 (18)	3 (17)	.92
Organisation	23 (82)	17 (94)	.23 ↑
Physical condition	17 (61)	13 (72)	.42 ↑
Psychological condition	18 (64)	7 (39)	.09 ↓
Therapy	22 (79)	16 (89)	.37 ↑
Complementary medicine	1 (4)	Undocumented	<.01
Patient preferences	4 (14)	Undocumented	.09
**Milestone conversation (MC 2):**	**N (%)** **51 (100)**	**N (%)** **47 (100)**	
Prognostic Awareness	16 (31)	22 (47)	.12 ↑
Advance Care Planning	13 (26)	6 (13)	.11 ↓
Organisation	28 (55)	30 (64)	.37 ↑
Physical condition	28 (55)	39 (83)	<.01 ↑
Psychological condition	35 (69)	24 (51)	.08 ↓
Therapy	50 (98)	36 (77)	<.01 ↓
Complementary medicine	4 (8%)	3 (6)	.78
Patient preferences	7 (14)	11 (23)	.22 ↑
**Milestone conversation (MC 3):**	**N (%)** **30 (100)**	**N (%)** **9 (100)**	
Prognostic Awareness	16 (53)	5 (56)	.91
Advance Care Planning	9 (30)	2 (22)	.65
Tumour progression	21 (70)	7 (78)	.65
Organisation	17 (57)	7 (78)	.25 ↑
Physical condition	20 (67)	7 (78)	.53 ↑
Psychological condition	23 (77)	5 (56)	.22 ↓
Therapy	29 (97)	6 (67)	<.01 ↓
Complementary medicine	1 (3)	Undocumented	.58
Patient preferences	8 (27)	1 (11)	.33 ↓
**Milestone conversation (ME 4):**	**N (%)** **2 (100)**	***n*** **= 1**	
Best supportive care	2 (100)	Undocumented	
Therapy (no further)	1 (50)	Undocumented	
**Other conversations**	**N (%)** **14 (100)**	N (%) **71 (100)**	
Prognostic Awareness	Undocumented	17 (23)	.05
Advance Care Planning	3 (21)	24 (32)	.41 ↑
Organisation	12 (86)	51 (69)	.20 ↓
Physical condition	8 (57)	48 (65)	.58
Psychological condition	7 (50)	38 (51)	.93
Therapy	9 (64)	36 (49)	.28 ↓
Complementary medicine	2 (14)	4 (5)	.23
Patient preferences	Undocumented	17 (23)	.05
**Conversations without ME number**	**N (%)** **9 (100)**	**N (%)** **26 (100)**	
Prognostic Awareness	3 (33)	5 (19)	.39 ↓
Advance Care Planning	2 (22)	4 (15)	.64
Organisation	9 (100)	15 (58)	.02 ↓
Physical condition	9 (100)	18 (69)	.06 ↓
Psychological condition	4 (44)	12 (56)	.93 ↑
Therapy	9 (100)	15 (58)	.02 ↓
Complementary medicine	1 (11)	3 (12)	.92
Patient preferences	1 (11)	5 (19)	.58

In the second assessment period (t2), 172 MCs were documented: 18 MC 1, 47 MC 2, 9 MC 3, and 1 MC 4. 42.3% of conversations (compared to 11% during t1) were not specified with an MC number: 26 other conversations without a special thematic focus and 71 conversations without documentation of the MC number (Table 2). There was no MC with a patient who was documented to be close to death. The mean duration of the MCs was 28 min (SD = 5.1; range: 5–50 min). The documentation of the routine records was not distributed equally among the nurse navigators. One of the nurse navigators documented a larger share than the two other nurse navigators.

### General topics

General topics were analysed for all types of MCs. Patients’ preferences were documented in all conversations up to a maximum of 27% (*n* = 8) (AP1) and 23% (*n* = 11) (AP2). The aspect “Organisation” was documented in 55–82% of the MCs during t1 and in 64–94% of the MCs during t2. Therapies were also written down in a majority of the MCs in both assessment periods (t1: 79–98%, t2: 67–89%). Psychological conditions were less often attended to during t2 (39–56%, t1: 64–77%). (Table 2).

### Milestone-specific topics

In MC 1, communication about diagnoses more often occurred during t2 (50% (*n* = 9), t1: 21% (*n* = 6)). Prognosis was recorded in less than 20% of the conversations (t1: 18% (*n* = 5), t2: 17% (*n* = 3)). For MC 2 and MC 3, the discussion of prognostic awareness and advance care planning was paramount. Prognostic awareness was more often documented in MC 3 (t1: 53% (*n* = 16), t2: 56% (n = 5)) than in MC 2 (t1: 31% (n = 16), t2: 47% (*n* = 22)). ACP was written down up to 30% (*n* = 9) of the conversations during t1 (MC 3, MC 2: 26% (*n* = 13)), but less often during t2 (MC 2: 13% (*n* = 6), MC 3: 22% (*n* = 2)). During t2, only one MC 4 was carried out. The documentation included no milestone-specific topics. (Table 2).

### Follow-up calls

During t1, 54 follow-up calls were conducted, which had a range of duration from five to 55 min. The average duration of follow-up calls was 19 min. During t2, 92 follow-up calls were documented, which had a range of duration from ten to 45 min. The average duration was 17 min.

In most follow-up calls, the physical condition of the patients was recorded (t1: 82% (*n* = 44), t2: 83% (*n* = 76)). Patients also occasionally brought up the topics of prognostic awareness (t1: 39% (*n* = 21), t2: 24% (*n* = 22)) and of advance care planning (t1:17% (*n* = 9), t2: 9% (*n* = 8)). (Table 3).

**Table 3:** Most documented topics of the follow-up calls – period comparison

Topics documented	t1	t2	***p***-value
**Total of records**	**N (%)** **54 (100)**	**N (%)** **92 (100)**	
**Physical condition**	**44 (82)**	**76 (83)**	**.86**
• General condition	• 18 (33)	• 33 (36)	**.76**
• Respiration	• 18 (33)	• 13 (14)	**<.01**
• Mobility	• 18 (33)	• 13 (14)	**<.01**
• Pain	• 18 (33)	• 22 (24)	**.22**
• Food & Beverarge	• 16 (30)	• 24 (26)	**.64**
• Skin & Hair	• 14 (26)	• 18 (20)	**.37**
• Sleep	• 9 (17)	• 8 (9)	**.15**
• Weight	• 5 (9)	• 1 (1)	**.02**
• Excretion	• 4 (7)	• 11 (12)	**.38**
• Nausea	• 2 (4)	• 5 (5)	**.64**
• Burden of symptoms	• Undocumented	• 7 (8)	**.04**
**Psychological condition**	**37 (69)**	**70 (76)**	**.32**
• Prognostic Awareness	• 21 (39)	• 22 (24)	**.06**
• Positiv feelings	• 18 (33)	• 28 (30)	**.72**
• Burden/excessive demand	• 8 (15)	• 18 (20)	**.47**
• Family/Friends	• 7 (13)	• 11 (12)	**.86**
• Sport as motivation	• 5 (9)	• 11 (12)	**.92**
• Relationship problems	• 5 (9)	• 7 (8)	**.73**
• Fears	• 3 (6)	• 7 (8)	**.64**
• Unrest	• 2 (4)	• 1 (1)	**.28**
• Listlessness	• 2 (4)	• 3 (3)	**.89**
**Medical care**	**39 (72)**	**60 (65)**	**.38**
• Oral medication	• 15 (28)	• 30 (33)	**.54**
• Chemotherapy	• 12 (22)	• 21 (23)	**.93**
• General therapy	• 8 (15)	• 11 (12)	**.59**
• Radiotherapy	• 6 (11)	• 11 (12)	**.88**
• Inpatient admission	• 6 (11)	• 3 (3)	**.06**
• Laboratory	• 5 (9)	• 7 (8)	**.73**
• CT	• 4 (7)	• 6 (7)	**.84**
• Rehabilitation	• 3 (6)	• 2 (2)	**.28**
• MRT	2 (4)	• 4 (4)	**.85**
• Medical operation	1 (2)	• Undocumented	**.19**
**Nursing care**	**13 (24)**	**16 (17)**	**.33**
• Home care/Social services	• 7 (13)	8 (9)	**.19**
• Care by family members	• 6 (11)	8 (9)	**.63**
• Self-supply	• Undocumented	3 (3)	**.18**
**Advance Care Planning**	**9 (17)**	**8 (9)**	**.15**
• SAPV	• 7 (13)	4 (4)	**.06**
• Patient decree	• 2 (4)	4 (4)	**.85**
• Precautionary power	• 1 (2)	1 (1)	**.70**
**Outpatient providers**	**6 (11)**	**11 (12)**	**.88**
• General practitioner	5 (9)	8 (9)	**.91**
• Psychological service	1 (2)	3 (3)	**.62**
• Radiation	Undocumented	1 (1)	**.44**
**Wishes/Hopes/Attitude to life**	**9 (17)**	**14 (15)**	**.82**
• Vacation	• 5 (9)	• 9 (10)	**.92**
• Improvement of health	• 4 (7)	• 4 (4)	**.43**
• Family events	• Undocumented	• 2 (2)	**.28**
**Appointments**	**30 (56)**	**52 (56)**	**.91**
**IPOS used**	**54 (100)**	**92 (100)**	
• Yes	22 (41)	42 (46)	
• No	32 (59)	50 (54)	

## Discussion

To assess to what extent the MCA is implemented in practice, a process evaluation was conducted. For an assessment of intervention fidelity, healthcare providers’ adherence to the manual regarding content of milestone conversations and follow-up calls was mixed. ACP was documented more often at January to May, while addressing prognostic awareness and documenting patient preferences were consolidated. Although in about half of the conversations these topics were not documented, we consider the documented conversations on key features of MCA as a major step towards its implementation as the communication concept was previously non-existent in this setting.

These results can be discussed in relation to multiple definitions of fidelity in the literature [21]. It provides a framework for discussing key features, but it is not necessary that each feature is included in all conversations as these need to be tailored to individual patient needs. According to Rietjens et al. [22], prognostic awareness is a complex issue with no internationally standardised form of survey or documentation. Furthermore, ACP lacks an international consensus on its definition. Nevertheless, in the MCA manual, existing recommendations that ACP in the palliative setting should be carried out as a communication process on the basis of individual readiness were included [22].

In a comparable study, the effectiveness of a nurse-led primary palliative care intervention was evaluated using visit checklists and visit audio-recordings to monitor adherence to intervention. Therefore, the intervention was divided into several clear defined components. For all components the fidelty was over 80% [23]. The difference with our study may be caused by a different method for assessing intervention fidelity, by the focus on routine clinical practice, by differences in the situation at baseline, or by other differences. Comparison of the studies is difficult. Another study using a systematic assessment procedure to report on implementation fidelity showed that 70% of all strategies were implemented with integrity [24]. However, this study focussed on the implementation strategy and not the intervention targeted at patients. In the MCA project the plan do-check-act-cycle was included as a measure to monitor implementation [7]. It was decided to rely on the routine clinical documentation to reflect the real-world setting as close as possible. This could be an explanation why it seems that fidelity remained on a lower level than in other studies as communication tends to be less well recorded than, for instance, test results and treatments. Future research of the concept may use a more rigorous monitoring of the intervention fidelity, such as direct observation or analysis of audiotaped conversations.

ACP and prognostic awareness were defined as specific topics of MC 2. The aspect of patient preferences was not tied to a particular MC. However, these key features are interdependent and important for every MC. Inaccurate prognostic awareness may hinder ACP and the consistency of care with patients’ goals [25]. Especially patients with advanced cancer need sufficient information for making realistic health-care decisions at the end of life. Studies have found a significant relationship between accurate prognostic awareness and reduced psychological distress in patients with advanced cancer [26]. Therefore, prognostic awareness takes on a high significance in the context of early palliative care. It has already been shown that well-informed patients express the wish for an early transition to palliative care in order to avoid aggressive therapies at the end of life [27]. The presence of prognostic awareness in turn increases the willingness to accept aspects of ACP. Conversely, ACP is an important strategy to improve end-of-life communication between patients and healthcare providers. Other potential benefits are that ACP allows patients to maintain a sense of control and gives them the opportunity to incorporate personal preferences [28].

Despite the relatively successful start of the implementation of the MCA, it should be considered how its implementation can be enhanced. The high emotional treshold in professionals for addressing these topics is well known [29]. Therefore, oncological societies emphasize the need to strenghten communication skills [30]. A communication training as this may be the initiation of a process that seeks for a change in behaviour and attitude. It seems likely that a longer follow-up would show higher fidelity [31]. On the other hand, a proportion of the trained health care professionals has been replaced by others as a consequence of planned rotations and other factors. It is a challenge to adapt the concept to practice while maintaining the three key features to all MCs which are important to support stage IV lung cancer patients.

Additionally, adherence to the MCA concept may be strenghtened by using a more rigorous plan to monitor adherence to the intervention and by providing support with additional training sessions and coaching for effective documentation. After a four-months training period with two theoretical and two practical on-the-job training sessions, only up to 50% of the relevant aspects are documented in the actual patient conversations. Furthermore, various conversations were documented as “Milestone conversations” without defining the precise phase in the disease trajectory. There might be several reasons which have to be further explored to respond accordingly.

Firstly, the performed training is only one aspect of dealing with relevant barriers to change in routine care. Implementation of the MCA concept meets with logistic challenges in a busy clinic setting. For instance, the physician and the nurse need to be available simultaniously as well as a protected room for the conversation. The role of local clinical opinion leaders seems crucial to keep the topic high on the agenda. Since behavioural change needs time and continuous support, “booster training sessions” might help to consolidate behaviour and knowledge (including phase definitions [32]) according to the MCA and facilitate routines [8]. Secondly, as indicated above, it is possible that topics were covered but not documented. Documentation by nurse navigators in communication is not standardized, but actually put down in plain free text. Out of the routine records now analysed, a documentation sheet could be developed to facilitate documentation. This might also foster interprofessional team communication and enhance good quality of patient care.

Thirdly, health care professionals may perceive milestone conversations as protected time with their patients, in which they are allowed to extend the usual consultation time. This might be a reason for the high percentage of “other conversations” after implementation of the MCA. Additionally, Milestone conversations stretched over multiple sessions, thus allowing for relationship building especially at the beginning of patient contact. Nevertheless, that could have led to a spread of content over multiple sessions and a fragmented documentation. The high number of aspects of physical condition documented may also be a sign for the respective high burden of this patient group and the priorities set by the patients concerning their needs.

Follow-up calls are seen as a useful tool to strengthen the key features of the concept. This way of communication serves not only to strengthen the relationship between patient and health care professionals, but also to clarify patient preferences. The possibility of having contact with the treatment team outside of planned MCs helps the patient to clarify his or her own status with regard to relevant information for ACP and prognostic awareness. Within the follow-up calls, nurse navigators provide emotional support to individuals tailored to patients’ unique needs. Furthermore, the follow-up calls offer the possibility to develop individual strategies that support the patient regarding prognostic awareness and ACP [33].

### Strenghts and limitations

The study is embedded in routine clinical practice and has therefore a high transferability of findings, although only one hospital was involved. The limitations of routine clinical documentation has already been highlighted. On the basis of the available documentation, we cannot assess contextual determinants of intervention fidelity [34]. The implementation activities heavily focused on training of health care professionals and logistic problems were addressed only after the first experiences. Finally, the data do not allow the analysis of individual patients’ pathways as the documented conversations could not be linked to individual patients. Patient relevant outcomes are evaluated within a current randomized controlled trial, though, and will be shortly available.

## Conclusion

The MCA provides a communication concept that oncologists and nurses can use to strengthen lung cancer patients’ attitudes towards prognostic awareness, ACP and patients preferences. The study shows that a major step towards its implementation in routine clinical practice was made, but also that room for further improvement exists. To support implementation of the concept, adjustments to the concept based on the process evaluation are needed. Further work should investigate factors that support and detract from intervention fidelity to improve it in the future.

## Supplementary information

**Additional file 1.** Checklist milestone conversations: quantitative developed checklist for the analysis of the MCs.

**Additional file 2.** Checklist for follow-up calls: quantitative developed checklist for the analysis of follow-up calls.

**Additional file 3.** Relevant term definitions MCs: Contains the definition of relevant terms from the checklist of MCs and explains them using a suitable example.

**Additional file 4.** Relevant term definitions Follow-up calls: Contains the definition of relevant terms from the checklist of Follow-up calls and explains them using a suitable example.

## Data Availability

The datasets used and analyzed during the current study are available from the corresponding author on reasonable request.

## Abbreviations

ACP: Advance Care Planning
IPOS: Integraded Palliative Outcome Scale
MC: Milestone Conversation
MCA: Milestone Communication Approach
