# Supplementary material for: Implementation of the milestones communication approach for patients with limited prognosis: evaluation of intervention fidelity
Source: BMC Palliat Care. 2020 Feb 18;19:21. doi: 10.1186/s12904-020-0527-1 (PMC7029500; doi:10.1186/s12904-020-0527-1)
Supplement: Supplementary file 1 — Additional file 1. Checklist milestone conversations: quantitative developed checklist for the analysis of the MCs. [file 12904_2020_527_MOESM1_ESM.docx]

| Performing physician | |  |  | Yes □ | No □ | |
| --- | --- | --- | --- | --- | --- | --- |
| Date | |  |  | Yes □ | No □ | |
| Present caregivers | |  |  | Yes □ | No □ | |
| Duration | n. a. □ | 10-20 □ | 20-30 □ | 40-50 □ | 50-60 □ | |
| Milestone conversation (ME) | n. a. □ | MC 1 □ | MC 2 □ | MC 3 □ | ME 4 □ | Other □ |
| Situation of disease | n. a. □ | Stable □ | Unstable □ | □ Debasing | Dying □ |  |
| **General topics** |  | **Organisation**  - Prescription  - Appointments  - Transport  - Aids and Offers  - Contact outpatient sector  - Requests  **Physical condition**  - General condition/Fatigue  - Mobility  - Nutrition  - Sleep  - Pain  - Adverse events  - Skin and Hair  - Burden of symptoms  - Excretion  **Psychological condition**  - Emotions  - Behaviour  - Reactions  **Advance Care Planning**  - Patient decree  - Precautionary power  - Shared decision making  - Care by family/friends  - SAPV/hospice  **Prognostic Awareness**  - Survival time  - Questions about disease  **Best Supportive Care**  - Palliative pain therapy  - Preferences regarding treatment  **Patient preferences**  - General wishes concerning privacy  - Hope  - Attitude to life  **Complementary medicine**  - Oil therapy  - Feet/foot reflex massage  - Mistletoe therapy  - Hyperthermia |  |  |  |  |
| Organisation | Yes □ | No □ |  |  |  |  |
| Physical condition | Yes □ | No □ |  |  |  |  |
| Psychological condition | Yes □ | No □ |  |  |  |  |
| Therapy | Yes □ | No □ |  |  |  |  |
| Complementary medicine | Yes □ | No □ |  |  |  |  |
| Patient preferences | Yes □ | No □ |  |  |  |  |
| **Milestone conversation I** |  |  |  |  |  |  |
| Diagnosis | Yes □ | No □ |  |  |  |  |
| Further diagnostics | Yes □ | No □ |  |  |  |  |
| Prognosis | Yes □ | No □ |  |  |  |  |
| Other topics |  |  |  |  |  |  |
| **Milestone conversation II** |  |  |  |  |  |  |
| Prognostic Awareness | Yes □ | No □ |  |  |  |  |
| Advance Care Planning | Yes □ | No □ |  |  |  |  |
| Other topics |  |  |  |  |  |  |
| **Milestone conversation III** |  |  |  |  |  |  |
| Tumour progression | Yes □ | No □ |  |  |  |  |
| Prognostic Awareness | Yes □ | No □ |  |  |  |  |
| Other topics |  |  |  |  |  |  |
| **Milestone conversation IV** |  |  |  |  |  |  |
| No therapy options | Yes □ | No □ |  |  |  |  |
| Best Supportive Care: | Yes □ | No □ |  |  |  |  |
| Other topics |  |  |  |  |  |  |

Additional file 1: Checklist milestone conversations
