# Supplementary material for: Implementation of the milestones communication approach for patients with limited prognosis: evaluation of intervention fidelity
Source: BMC Palliat Care. 2020 Feb 18;19:21. doi: 10.1186/s12904-020-0527-1 (PMC7029500; doi:10.1186/s12904-020-0527-1)
Supplement: Supplementary file 2 — Additional file 2. Checklist for follow-up calls: quantitative developed checklist for the analysis of follow-up calls. [file 12904_2020_527_MOESM2_ESM.docx]

| IPOS used  Duration  Date given | Yes □  10-20□  Yes □ | No□  20-30□  No□ | 40-50□ |  | 50-60□ | |  |
| --- | --- | --- | --- | --- | --- | --- | --- |
| **Organisation** | Appointments | | | Yes □ | | No□ | |
| **Physical condition** | General condition/Fatigue | | | Yes □ | | No□ | |
|  | Mobility | | | Yes □ | | No□ | |
|  | Nutrition and Beverages  Skin & Hair | | | Yes □  Yes □ | | No□  No□ | |
|  | Weight | | | Yes □ | | No□ | |
|  | Excretion | | | Yes □ | | No□ | |
|  | Breathing | | | Yes □ | | No□ | |
|  | Cough (with/without sputum) | | | Yes □ | | No□ | |
|  | Burden of symptoms | | | Yes □ | | No□ | |
|  | Sleep | | | Yes □ | | No□ | |
|  | Pain | | | Yes □ | | No□ | |
|  | Nausea | | | Yes □ | | No□ | |
| **Psychological condition** | Fears | | | Yes □ | | No□ | |
|  | Unrest | | | Yes □ | | No□ | |
|  | Listlessness | | | Yes □ | | No□ | |
|  | Burden/excessive demand | | | Yes □ | | No□ | |
|  | Relationship problems | | | Yes □ | | No□ | |
|  | Positive feelings | | | Yes □ | | No□ | |
|  | Family/Friends as a support | | | Yes □ | | No□ | |
|  | Sport as motivation | | | Yes □ | | No□ | |
|  | Prognostic Awareness | | | Yes □ | | No□ | |
| **Medical care** | Medication | | | Yes □ | | No□ | |
|  | General therapy | | | Yes □ | | No□ | |
|  | Radiation | | | Yes □ | | No□ | |
|  | Immunotherapy | | | Yes □ | | No□ | |
|  | Chemotherapy | | | Yes □ | | No□ | |
|  | CT | | | Yes □ | | No□ | |
|  | MRT | | | Yes □ | | No□ | |
|  | Medical operation | | | Yes □ | | No□ | |
|  | Blood sampling, Laboratory | | | Yes □ | | No□ | |
|  | Inpatient admission | | | Yes □ | | No□ | |
|  | Rehabilitation | | | Yes □ | | No□ | |
| **Nursing care** | Care by family members | | | Yes □ | | No□ | |
|  | Home care/Social services | | | Yes □ | | No□ | |
|  | Self-supply | | | Yes □ | | No□ | |
| **Advance Care Planning** | SAPV  Patient decree  Precautionary power | | | Yes □  Yes □  Yes □ | | No□  No□  No□ | |
| **Outpatient providers** | General practitioner | | | Yes □ | | No□ | |
|  | Outpatient psychological service | | | Yes □ | | No□ | |
|  | Outpatient radiation | | | Yes □ | | No□ | |
| **Wishes/Hopes/Attitude to life** | Improvement of health | | | Yes □ | | No□ | |
|  | Vacation | | | Yes □ | | No□ | |
|  | Family events | | | Yes □ | | No□ | |

Additional file 2: Checklist for follow-up calls
