# Supplementary material for: Implementation of the milestones communication approach for patients with limited prognosis: evaluation of intervention fidelity
Source: BMC Palliat Care. 2020 Feb 18;19:21. doi: 10.1186/s12904-020-0527-1 (PMC7029500; doi:10.1186/s12904-020-0527-1)
Supplement: Supplementary file 3 — Additional file 3. Relevant term definitions MCs: Contains the definition of relevant terms from the checklist of MCs and explains them using a suitable example. [file 12904_2020_527_MOESM3_ESM.docx]

| **Terms** | | **Definition** | **Example** | |
| --- | --- | --- | --- | --- |
| **Organization** | | | | |
| Prescribes | | Doctor’s prescription authorizing a patient to receive a medication or treatment. | Prescription for Targin, Zolpidem, food supplements | |
| Appointments | | Refers to all internal and external appointments. | Appointments for diagnostic, inpatient admissions | |
| Transport | | Concerning the organization and information of patient transport | Issue of transport tickets | |
| Aids and Offers | | Organisation and information of aids and services for patients to support daily life activities | Wheelchair, shower stool, nursing bed | |
| Contact outpatient sector | | Includes aspects related to contact with the outpatient sector to strengthen the supply network. SAPV is excluded because this aspect is assigned to advance care planning. | Outpatient nursing service, contact GP or other specialists | |
| Requests | | Includes application formalities and aims to obtain a specific social or health benefit | Assistance with the application for care level or disabled pass | |
| **Physical conditions** | | | | |
| General condition/Fatigue | | General condition/Fatigue in humans is extreme tiredness and weakness arising from mental or physical effort [35] | Reduced general condition, manifests itself in reduced condition | |
| Mobility | | Refers to all aspects and conditions related to mobility that are important for the patient in the context of the disease. Also refers to diseases of the musculoskeletal system | Patient states that he has limited mobility in everyday life, making it difficult to climb stairs in particular | |
| Nutrition and Beverage | | Refers to all aspects regarding food and beverage and their effects | Appetite, loss of appetite, weight gain, weight loss, change of diet, special type of diet | |
| Sleep | | Refers to all sleeping habits which are discussed in the MCs | Problems falling asleep, problems sleeping through, good sleep without problems | |
| Pain | | Relates to unpleasant physical sensations caused by cancer disease or comorbidities | Patient complains of thoracic pain or bone pain. | |
| Adverse events | | This refers to all undesirable events that occur during oncological treatment. | Side effects such as nausea and vomiting,  Swelling of extremities, complication following blood transfusion | |
| Skin and Hair | | Covering aspects of problems, questions, occurrences related to skin and hair | Hair loss, wig, dry skin, rash of the skin | |
| Burden of symptoms | | Burden of symptoms is defined as the subjective, quantifiable prevalence, frequency, and severity of symptoms that represent a physiological burden on patients and elicit multiple negative, physical, and emotional responses from patients [36] | Patients explicitly state in the MCs that their symptoms became better/worse in the course of the oncological disease. | |
| Excretion | | Refers to human excreta and includes both general habits and existing problems in treatment | Constipation, diarrhea, digestive problems, urinary tract infection | |
| **Psychological conditions** | | | | |
| Emotion | | Feelings, moods, affects the patient/relatives shows during MCs | Joy, sorrow, trust, anxiety, anger | |
| Behaviour | | Refers to the visible actions and reactions of patients/relatives during MCs emanating from the disease  But it also concerns the conscious decision to behave in a certain way. | Aggressive, disinterested, interested, shy    Quitting smoking, preparing for therapy through certain behaviours | |
| Reaction | | Acts/reactions as a result of notifications by doctor/caregiver. | Patient is very desperate and disintegrated after notification of diagnosis | |
| **Advance Care Planning:** enables patients to make plans about their future health care | | | | |
| Patient decree | Patient decree regulates medical treatment in the final stage of an illness or following a serious accident [37] | | Patient addresses the patient decree on his own initiative or wishes to obtain information on this subject | |
| Precautionary power | With a precautionary power, which is regularly drawn up in writing, the patient gives one or more persons the power of representation to make legally binding decisions for him (e.g. in the field of health care); the patient agrees with his representative to make use of the power of attorney in the event of a future incapacity for business or inability to consent [38] | | Patient reports on his own the existence of a precautionary power or would like to be informed about it. | |
| Shared decision making | Participatory decision-making, which means that patients should actively participate in the decision-making process. To encourage patient participation, the physician should provide evidence when summarizing the issues at hand and trying to explain the pros and cons of each option. In other words, patient preferences should be taken into account during treatment [39].  It is also necessary that there is interaction with the doctor/nurse and that a joint therapy decision is being discussed. The sole expression of a preference belongs to "prognostic awareness” | | Patients express their preferences within the MCs, such as rejecting certain therapies. | |
| Care by family/friends | The care of the patient is guaranteed by the family network, which means that there is little or no need for external care (e.g. by the nursing service). | | Patient lives with children in the same house or relatives live in close proximity and visit daily to support the patient. | |
| - SAPV/hospice | 1. Special outpatient palliative care and home providing care for the sick or terminally ill. | | patient was informed about the possibility of SAPV or already uses this service or patient wants to spend the last phase of his life in the hospice | |
| **Prognostic Awareness** | | | | |
| Survival time | | Awareness of terminal prognosis and shortened life expectancy | | Patients ask the treatment team how long they will still live |
| Questions about disease | | Patients inquire about the extent of the disease and would like to receive information on the current situation | | The following questions are typical examples:  Where did I get metastases?  What is the best therapy for my illness?  Has the tumour already shrunk as a result of chemotherapy? |
| **Best supportive care** | | | | |
| Palliative pain therapy | Palliative pain therapy aims to alleviate the symptoms of a serious illness and to achieve the best possible quality of life. The treatment does not fight the disease, but improves its consequences. | | | Patients complain of increasing pain and express the wish for an adapted  Pain therapy (Increasing the dose of opiates) |
| Preferences regarding treatment | All therapy wishes of the patient, which contribute to the improvement of quality of life. | | | Wish for additional painkiller, Rejecting certain medications |
| **Patient preferences** | | | | |
| General wishes concerning privacy | Refers to requests outside treatment that patients express in the course of MCs. These wishes therefore only concern private life. | | | Patient reports on vacation plans, birthday celebrations, birth of grandchildren |
| Hopes | Hopes of patients in the context of the disease | | | Patients hope for improvement of the disease |
| Attitudes to life | The attitude to life expresses what is important for the patient in life and how it influences his illness. | | | Patients said that they will take things as they come along or patients express a strong will to survive |
| **Complementary medicine:** adapted as additional methods in oncology | | | | |
| Oil therapy | This includes both aroma oil therapies and the method of oil extraction.  Aroma oil therapy: In aroma therapy, essential oils of medicinal plants are used as fragrances. The efficacy of aroma therapy in a range of physical and psychological symptoms is evidence-based [40].  Oil extraction: originates from Ayurveda medicine. For about ten minutes (or longer) a tablespoon of sunflower, sesame or olive oil should be moved in the mouth and between the teeth. As the oil contains the toxins and harmful bacteria of the mouth and throat, it should be spat out after the oil has been extracted | | | Patients want the treatment team's opinion on aroma oil therapy or oil extraction. Patients report on their experiences with the two complementary medicine methods |
| Feet/foot reflex massage | Holistic method of complementary medicine. The foot reflex zone massage is based on the idea that energies circulate between the organs and aims to release blockages. Is effective in reducing pain levels and incidence of distension [41]. | | | Patients ask for contact or report on the effects of the foot reflex massage. |
| Mistletoe therapy | Mistletoe therapy is one of the most commonly used complementary methods to improve the quality of life and reduce the side effects caused by systemic therapy in cancer treatments. Furthermore, mistletoe extracts has an impact on survival [42]. | | | Patients mention mistletoe therapy in the MCS and report where they perceive it and how they feel about the treatment. |
| Hyperthermia | Treatment procedure in which body areas are brought to a controlled temperature of up to 43°C for about one hour. This is usually done by electromagnetic waves, which are guided into the tumour from the outside via antenna-like applicators. Hyperthermia treatment is particularly suitable for large inoperable tumours or advanced disease [43] | | | Patients request information on hyperthermia or contact details. Patients talk about their experiences and where they perceived the therapy. |

Additional file 3: Relevant term definitions MCs
