# Supplementary material for: Implementation of the milestones communication approach for patients with limited prognosis: evaluation of intervention fidelity
Source: BMC Palliat Care. 2020 Feb 18;19:21. doi: 10.1186/s12904-020-0527-1 (PMC7029500; doi:10.1186/s12904-020-0527-1)
Supplement: Supplementary file 4 — Additional file 4. Relevant term definitions Follow-up calls: Contains the definition of relevant terms from the checklist of Follow-up calls and explains them using a suitable example. [file 12904_2020_527_MOESM4_ESM.docx]

| **Terms** | **Definition** | **Example** | |
| --- | --- | --- | --- |
| **Organization** | | | |
| Burden of symptoms |  | |  |
| Appointments | Refers to all internal and external appointments. | Appointments for diagnostic, inpatient admissions | |
| **Physical condition** | | | |
| General condition/Fatigue | General condition/Fatigue in humans is extreme tiredness and weakness arising from mental or physical effort [35] | Reduced general condition, manifests itself in reduced condition | |
| Mobility | Refers to all aspects and conditions related to mobility that are important for the patient in the context of the disease. Also refers to diseases of the musculoskeletal system | Patient states that he has limited mobility in everyday life, making it difficult to climb stairs in particular | |
| Nutrition and Beverages | Refers to all aspects regarding food and beverage and their effects | Appetite, loss of appetite, weight gain, weight loss, change of diet, special type of diet | |
| Skin & Hair | Covering aspects of problems, questions, occurrences related to skin and hair | Hair loss, wig, dry skin, rash of the skin, Visit wig studio | |
| Weight | Covers all aspects of weight and refers to changes, problems as well as normality’s | Weight loss, weight gain, constant weight during therapy | |
| Excretion | Refers to human excreta and includes both general habits and existing problems in treatment | Constipation, diarrhea, digestive problems, urinary tract infection | |
| Breathing | Refers to aspects of breathing and covers changes, problems as well as normalities. | Dyspnea and therefore oxygen demand | |
| Cough (with/without sputum) | A cough is a sudden expiratory movement produced by the respiratory muscles which is a reaction to irritation of the respiratory tract. The cause of the irritation can be caused in many ways | Patient suffers from persistent cough with sputum | |
| Temperature | This includes all aspects related to the regulation of body temperature. This includes all aspects related to the regulation of body temperature and includes problems, changes and normalities. | Patient complains of fever or the feeling of hypothermia | |
| Sleep | Refers to all sleeping habits which are discussed in the MCs | Problems falling asleep, problems sleeping through, good sleep without problems | |
| Pain | Relates to unpleasant physical sensations caused by cancer disease or comorbidities | Patient complains of thoracic pain or bone pain. | |
| Nausea | Nausea is a feeling of sickness usually projected onto the gastrointestinal tract that can be associated with vomiting. | Patient suffers from nausea after chemotherapy, Patient reports vomiting. | |
| Burden of symptoms | Burden of symptoms is defined as the subjective, quantifiable prevalence, frequency, and severity of symptoms that represent a physiological burden on patients and elicit multiple negative, physical, and emotional responses from patients [36] | Patients explicitly state in the MCs that their symptoms became better/worse in the course of the oncological disease. | |
| **Psychological condition** | | | |
| Fears | Refers to any kind of anxiety reported by the patient or approached by the treatment team. | Fear of death, fear of the future, fear of further disease progression, financial fears | |
| Unrest | Restlessness caused by the disease and the living conditions it causes. | Patient expresses inner restlessness | |
| Listlessness | All these aspects indicate that the patient does not feel a desire/not an impulse for the most different things of daily life | Patient has no interest to pursue previous hobby or patient is motiveless in relation to household tasks | |
| Burden/excessive demand | This includes all points that burden the patient and are usually caused by the disease. | Patient talks about high burden, which he feels from illness | |
| Relationship problems | Refers to problems between at least two people (both within and outside the family). | Marriage problems or problems with children, because they cannot accept the patient's attitude towards the disease. | |
| Positive feelings | Includes all positive feelings expressed by the patient during the follow-up sessions and which contribute to a positive reinforcement of the disease. | Joy about decline of tumor  patient is happy that hair regrowth again | |
| Family/friends as a support | To enjoy the contact to family members or close friends is an essential strategy for coping with the disease situation | Weekly visit of children, meeting with friends for a walk | |
| Sport as motivation | All aspects that include the habits regarding physical activity as motivation | Weekly yoga course or daily one-hour walk | |
| Prognostic awareness | Awareness of terminal prognosis and shortened life expectancy,  Patients inquire about the extent of the disease and would like to receive information on the current situation | During the follow-up session, patients express that they know about the terminal disease. | |
| **Medical care** | | | |
| Medication | Medicinal products are in particular substances and preparations of substances which, when used in human beings, are intended to cure, alleviate, prevent or detect diseases, suffering, bodily harm or discomfort, and substances which are used for diagnosis or influence mental states [44] | Medicines for nausea, pain, dose adjustment | |
| General therapy | Refers to all aspects of general treatment that have no special focus | Patient asks whether changes in the course of therapy are planned | |
| Radiation | Concerns all aspects related to the topic of radiation. Radiotherapy is one of the central pillars of cancer therapy alongside surgery and chemotherapy | Next appointments for radiation, patient reported on radiation | |
| Immunotherapy | The immune system plays a central role in the prevention of tumour diseases, including the specific recognition and elimination of tumour cells by means of tumour-specific antigens that express them or molecules induced by cellular stress. Thus, both mechanisms of innate and adaptive immune defence are involved [45] | Patient asks if immunotherapy is suitable for him or patient reports from personal experience with immunotherapy | |
| Chemotherapy | Cancer chemotherapy refers to the administration of cytotoxic chemicals, e.g. chemicals with cell-killing properties, with the aim of eliminating the tumour or reducing the tumour [46] | Appointments, Patient reports on personal experiences with chemotherapy | |
| CT | Diagnostic apparatus using X-rays | Appointments | |
| MRT | Diagnostic apparatus employing strong magnetic field and radio waves | Appointments | |
| Medical operation | Surgical intervention | Appointments, Regeneration after surgery | |
| Blood sampling, Laboratory | usually capillary blood collection to determine certain parameters | Results of the last blood sampling, | |
| Inpatient admission | Staying of a patient in hospital through the implementation of targeted diagnostic and therapeutic measures | Inpatient chemotherapy, inpatient admission because of deterioration of the state of health | |
| Rehabilitation | Rehabilitation services include all medical services which serve to prevent, eliminate, reduce or compensate for a disability or need for care, to prevent its worsening or to mitigate its consequences. | Patient talks about forthcoming rehab and expresses feelings about it | |
| **Nursing care** | | | |
| Care by family members | The care of the patient is guaranteed by the family network, which means that there is little or no need for external care (e.g. by the nursing service). | Patient lives with children in the same house or relatives live in close proximity and visit daily to support the patient. | |
| Home care/Social services | Includes the use of nursing services, with the exception of SAPV. | Every morning the patient is helped to dress by a nursing service or the patient inquiries about the possibilities of an outpatient nursing service. | |
| Self-supply | Patient is able to carry out all activities of daily life at home independently. The use of help from family members or a nursing service is not necessary. | Patient can wash and dress independently, patient is mobile | |
| **Advance Care Planning** | | | |
| SAPV/hospice | Special outpatient palliative care and home providing care for the disease or terminally illness. | Patient was informed about the possibility of SAPV or already uses this service or patient wants to spend the last phase of his life in the hospice | |
| Patient decree | Patient decree regulates medical treatment in the final stage of an illness or following a serious accident [37] | Patient addresses the patient decree on his own initiative or wishes to obtain information on this subject | |
| Precautionary power | With a precautionary power, which is regularly drawn up in writing, the patient gives one or more persons the power of representation to make legally binding decisions for him (e.g. in the field of health care); the patient agrees with his representative to make use of the power of attorney in the event of a future incapacity for business or inability to consent [38] | Patient reports on his own the existence of a precautionary power or would like to be informed about it. | |
| **Outpatient providers** | | | |
| General practitioner | A doctor who provides general medical treatment in the outpatient sector. | Weekly blood collection by GP, exchange between GP and clinic physician | |
| Outpatient psychological service | outpatient psychologists who support the patient in the course of the illness | Patient reports on weekly therapy session, asks for contact addresses | |
| Outpatient radiation | Outpatient radiotherapy as a component of cancer therapy. | Patient names appointment for next outpatient radiation treatment | |
| **Wishes/Hopes** |  |  | |
| Improvement of health | Patient hopes for improvement of underlying disease and/or associated symptoms | Patient hopes for tumor reduction or improvement of pain situation | |
| Vacation | Time period outside of everyday life, which serves for the recovery | Patient wants to see the sea again | |
| Family events | Upcoming family events, which may take place in different forms | Patient reports on vacation plans, birthday celebrations, birth of grandchildren | |

Additional file 4: Relevant term definitions follow-up-calls
